# Composite Mineralized Collagen/Polycaprolactone Scaffold-Loaded Microsphere System with Dual Osteogenesis and Antibacterial Functions

**DOI:** 10.3390/polym16172394

**Published:** 2024-08-23

**Authors:** Yuzhu He, Qindong Wang, Yuqi Liu, Zijiao Zhang, Zheng Cao, Shuo Wang, Xiaoxia Ying, Guowu Ma, Xiumei Wang, Huiying Liu

**Affiliations:** 1 School of Stomatology, Dalian Medical University, Dalian 116044, China; yuzhuh0723@dmu.edu.cn (Y.H.); wqd820343043@163.com (Q.W.); lyq19940508@163.com (Y.L.); 948233717zhangzijiao@gmail.com (Z.Z.); yingxiaoxia1103@163.com (X.Y.); lhy04512000@dmu.edu.cn (H.L.); 2 Academician Laboratory of Immune and Oral Development & Regeneration, Dalian Medical University, Dalian 116044, China; 3 State Key Laboratory of New Ceramics and Fine Processing, School of Materials Science and Engineering, Tsinghua University, Beijing 100084, China; 1988caozheng@163.com (Z.C.); 13811972935@163.com (S.W.)

**Keywords:** scaffolds, composite microspheres, antimicrobial peptide, antibacterial, osteogenesis

## Abstract

Biomaterials play an important role in treating bone defects. The functional characteristics of scaffolds, such as their structure, mechanical strength, and antibacterial and osteogenesis activities, effectively promote bone regeneration. In this study, mineralized collagen and polycaprolactone were used to prepare loaded porous scaffolds with bilayer-structured microspheres with dual antibacterial and osteogenesis functions. The different drug release mechanisms of PLGA and chitosan in PLGA/CS microspheres caused differences in the drug release models in terms of the duration and rate of Pac-525 and BMP-2 release. The prepared PLGA_(BMP-2)_/CS_(Pac-525)_@MC/PCL scaffolds were analyzed in terms of physical characteristics, bioactivity, and antibacterial properties. The scaffolds with a dimensional porous structure showed similar porosity and pore diameter to cancellous bone. The release curve of the microspheres and scaffolds with high encapsulation rates displayed the two-stage release of Pac-525 and BMP-2 over 30 days. It was found that the scaffolds could inhibit *S. aureus* and *E. coli* and then promote ALP activity. The PLGA_(BMP-2)_/CS_(Pac-525)_@MC/PCL scaffold could be used as a dual delivery system to promote bone regeneration.

## 1. Introduction

Bone replacement surgery is the main therapy used for fracture, tumors, trauma and skeletal infections [1]. The autograft and allograft are the two main approaches for bone repair, which have shortcomings include site morbidity, insufficient donors, immune rejection, etc.; as such, the artificial biomaterials that are being widely used for bone defect clinical treatment deserve intensive researched [2]. Bone scaffolds are designed to have multiple characteristics, such degradability, porousness, mechanical strength, biocompatibility, and certain biofunctions, which provide the space and stimulation for the proliferation and differentiation of osteocytes [3]. Polycaprolactone (PCL), a synthetic biodegradable polymer with high initial mechanical strength and a slow degradation rate, is much easier to fabricate and can be molded into desirable shapes and sizes according to the requirements [4,5]. PCL has been processed into various tissue-engineered materials, including being 3D-printed into scaffolds and electrospun into sponges and membranes with PEG [6,7,8]. Mineralized collagen (MC) is recognized as an excellent material for promoting osteogenesis, but it lacks sufficient mechanical strength [9,10,11,12,13,14]. A PCL matrix containing dispersed MC could constitute an appropriate scaffold for the attachment, proliferation and osteogenic differentiation of mesenchymal stem cells (MSCs) [15].

Additionally, the functional modification of scaffolds by loading bioactive drugs as well as growth factors, stem cells, and antibiotics using advanced technology can improve the osteogenesis, anti-inflammation, and antibacterial abilities of the scaffolds [15]. The capability of scaffolds to maintain long-term antibacterial activity is beneficial for treating infective bone defects. Pac-525 (Ac-Lys-Trp-Arg-Arg-Trp-Val-Arg-Trp-Ile-NH_2_), a synthetic amino acid sequence, has been confirmed to have wide-spectrum antibacterial activity and low drug resistance [16,17]. The Val, Ile, Phe, and Trp contained in Pac-525, as rich hydrophobic amino acids, could aid in their insertion deep into the interior of lipopolysaccharide (LPS). The other basic amino acids such as the Lys and Arg in Pac-525 would help neutralize LPS due to their close packing with the phosphate groups or saccharides of LPS [18]. Therefore, the antibacterial effects of Pac-525 could avoid the development of bacterial resistance, which is an issue with traditional antibiotics. Bone morphogenetic protein-2 (BMP-2) is one of the most important osteogenic growth factors, having biological activities in osteogenesis and proliferation [19,20]. The application of Pac-525 and BMP-2 could allow scaffolds to have dual osteogenesis and antibacterial functions.

It is worth noting that bone regeneration is a long-term process. Considering the situation regarding low local concentration and metabolic loss in vivo, it is necessary to select an appropriate carrier for the local long-term release of drugs [21,22]. The present materials used for carriers include natural organic materials, organic polymer materials, inorganic nonmetallic materials, etc. Poly(lactic-co-glycolic acid) (PLGA) is a biodegradable polymer with a long degradation process of up to three months, which helps BMP-2 achieve a long-term osteogenic effect [23,24]. Although PLGA shows good biocompatibility and is not cytotoxic, its degradation-induced weak acidic environment poses the potential risk of local inflammation [25]. Chitosan (CS) is obtained from the partial deacetylation of chitin, the aqueous solution of which is weakly alkaline, which can balance the local acid–base environment [26]. A drug carrier prepared with CS releases Pac-525 in the short term, protecting the bone defect from infection before the release of BMP-2 [27]. Therefore, the particles prepared with PLGA and CS could achieve the dual-stage release of BMP-2 and Pac-525 while maintaining biological function. The primary stage of Pac-525 release can guarantee sterile conditions at the site of the bone defect, and the secondary release of BMP-2 can produce a long-term promoting effect on the bone tissue regeneration process.

In this research, we successfully fabricated PLGA_(BMP-2)_/CS_(PAC-525)_ composite microspheres through electrospraying and crosslinking emulsion methods. The composite microspheres, which exhibited a sphere-in-sphere structure, were assembled in MC/PCL scaffolds to enhance their osteogenic and antibacterial functions. Finally, we investigated the release behavior, physical characteristics, biological viability, and antibacterial capability of the scaffolds, demonstrating their potential for bone regeneration.

## 2. Materials and Methods

### 2.1. Materials

BMP-2 (Invitrogen, Carlsbad, CA, USA) and antimicrobial peptide Pac-525 (Ac-KWRRWVRWI-NH2) were synthesized by Qiangyao Bio-Technology Co. Ltd. (purity > 95%, Shanghai, China). Poly (lactic-co-glycolic acid) (PLGA, 75:25, Mw ≈ 50 kDa, Medical Equipment Research Institute, Jinan, China) and polycaprolactone (PCL, Mw ≈ 3 × 10^5^) were purchased from the Medical Equipment Research Institute (Jinan, China). Chitosan (Mw: 1 × 10^5^–3 × 10^5^) was purchased from Bailingwei Science and Technology Co. Ltd. (Beijing, China). All other chemicals used were obtained from Chemical Reagent Co, Ltd. (Beijing, China).

### 2.2. Preparation

#### 2.2.1. PLGA_(BMP-2)_ Microspheres

PLGA microspheres loaded with BMP-2 (PLGA_(BMP-2)_) were prepared by electrospraying. Briefly, a certain amount of PLGA was dissolved in methylene chloride under magnetic stirring, obtaining a 60 mg/mL solution. Then, 0.5 mg/mL BMP-2 solution was mixed with PLGA-methylene chloride solution at a volume ratio of 1:40. The mixture was transformed into a water-in-oil (W/O) emulsion through sonication (Scientz-IID, Ningbo Science Biotechnology Co., Ltd., Ningbo, China) on ice at a power of 200 W for 30 s. Subsequently, the emulsion solution was sprayed at a constant flow rate of 1 mL/h using a micro-infusion pump. The voltage applied to the nozzle tip was 6 kV, and the distance between the nozzle tip to the aluminum collection plate was 20 cm. Finally, the PLGA_(BMP-2)_ microspheres were freeze-dried for 24 h to remove the residual solvent.

#### 2.2.2. PLGA_(BMP-2)_/CS_(Pac-525)_ Composite Microspheres

PLGA_(BMP-2)_/CS_(Pac-525)_ composite microspheres were prepared using the crosslinking emulsification method. Firstly, CS was dissolved in an aqueous acetic acid solution at a dose of 3% (*w*/*v*). The prepared PLGA_(BMP-2)_ microspheres and Pac-525 were dispersed in the chitosan solution and stirred magnetically at room temperature. Then, the above mixture was poured into 60 mL of liquid paraffin containing 1.8 mL of Span-80 to form a W/O emulsion, which was stirred magnetically for 30 min at room temperature. Thereafter, a 5% (*w*/*v*) aqueous sodium tripolyphosphate (TPP) solution was slowly added to the emulsion to solidify the microspheres. After settling overnight, the obtained PLGA_(BMP-2)_/CS_(Pac-525)_ composite microspheres were alternately douched with excess amounts of petroleum and isopropyl alcohol three times to clean off the remaining liquid paraffin and Siban-80. Lastly, PLGA_(BMP-2)_/CS_(Pac-525)_ composite microspheres were lyophilized for 24 h after washing with water. The obtained PLGA/CS microspheres were stored at −20 °C until use. Fluorescein-labeled Nile red (MedChemExpress, Monmouth Junction, NJ, USA) and FITC (Biofroxx, Einhausen, Germany) were used to replace BMP-2 and Pac-525, respectively, in PLGA _(Nile)_/CS _(FITC)_ composite microspheres with the above methods.

#### 2.2.3. PLGA_(BMP-2)_/CS_(Pac-525)_@MC/PCL Scaffolds

Briefly, PCL powder with a molecular weight of 3 × 10^5^ was dissolved in dioxane at a final concentration of 10% (*m*/*v*) and stirred gently at room temperature. MC powder was added to the stirred PCL solution at a weight ratio of 1:1 (MC: PCL). After that, the mixture was added into the pores (10 mm diameter and 20 mm height) of the polytetrafluoroethylene mold. Then, 100 mg of the obtained PLGA_(BMP-2)_/CS_(Pac-525)_ composite microspheres was added into the empty space and mixed thoroughly. Finally, the mold containing the samples was placed in a refrigerator at −20 °C overnight and then freeze-dried for 48 h to remove the dioxane, resulting in a pure PLGA_(BMP-2)_/CS_(Pac-525)_@MC/PCL scaffold.

### 2.3. Characterization of the Physical and Chemical Properties

#### 2.3.1. Morphology

The morphology of the PLGA_(BMP-2)_/CS_(Pac-525)_ microspheres and PLGA_(BMP-2)_/CS_(Pac-525)_@MC/PCL scaffolds was observed using field-emission scanning electron microscopy (SEM, Merlin Zeiss, Jena, Germany). Microspheres and broken microspheres were dispersed in ethyl alcohol and dropped onto a metal platform for observation. The scaffolds were broken for SEM observation. All the samples were coated with a layer of gold and observed at an accelerating voltage of 10 kV. The fluorescein-labeled PLGA_(BMP-2)_/CS_(Pac-525)_ microspheres were observed using confocal laser scanning microscopy (CLSM, Zeiss LSM710, Jena, Germany). The size distribution of the microspheres and scaffolds was measured using a Nano Measurer software v1.2.

#### 2.3.2. Encapsulation Rates of Microspheres

The drug encapsulation rates of the PLGA_(BMP-2)_/CS_(Pac-525)_ microspheres were tested as follows: 20 mg of PLGA/CS microspheres was completely dissolved in a 2% acetic acid solution and then centrifuged at 5000 rpm for 15 min to separate the supernatant (Sp). The precipitate was dried and weighed and then dissolved in acetonitrile (0.7 mL) and 0.01 mol hydrochloric acid buffer (1.3 mL) while stirring. The resulting supernatant (Sb) was centrifuged at a speed of 5000 rpm for 15 min. The concentrations of Pac-525 in Sp and BMP-2 in the Sb were determined using a UV–VIS spectrophotometer (Thermo Fisher Scientific, Waltham, MA, USA) (280 nm) and a BMP-2 ELISA kit (Abcam, Cambridge, UK), respectively. The determination for each sample group was repeated three times. And the encapsulation rate was calculated using the following formula:

(1) $$Encapsulationefficiency(\%)=\frac{Amountofdruginmicrospheres}{Totalamountofdrug}\times 100\%$$

#### 2.3.3. Porosity of PLGA_(BMP-2)_/CS_(Pac-525)_@MC/PCL Scaffolds

The porosity of the scaffold was measured using the fluid displacement method with water (ρ = 1 g/cm^3^). Briefly, a sample with a known mass (M1) was placed into a suction bottle containing a certain volume of water. The air from the bottle was then extracted, allowing water to replace the air within the scaffold. Afterwards, the sample was removed, the water on the surface of the scaffold was quickly dried, and the mass (M2) was recorded. The porosity of the scaffold (*P*) is represented as

(2) $$P=\frac{M2-M1}{\rho V}\times 100\%$$

#### 2.3.4. Compressive Strength of PLGA_(BMP-2)_/CS_(Pac-525)_@MC/PCL Scaffolds

The compressive strength of the scaffolds was tested using a universal mechanical machine (SHIMADZU AG-IC, Kyoto, Japan) with a 10 kN load cell, applying a compression rate of 0.5 mm/min. The force was applied until the scaffolds underwent a vertical deformation of 30%. Starting from the 20% strain point, the compressive strength was determined by drawing a straight line with the same slope as the elastic modulus and intersecting the curve. The test for each sample group was repeated three times.

#### 2.3.5. Drug Release Test in Vitro

The release behavior of the BMP-2 and Pac-525 from the microspheres and scaffolds was determined as follows: 100 mg of microspheres was soaked in 2 mL of PBS in a shaker at 37 °C. At certain time points, 1 ml of supernatant was collected and supplemented with 1 mL of fresh PBS. For the scaffolds, the scaffolds containing 100 mg of microspheres were placed in 3 mL (V_0_) of PBS and shaken at 37 °C. At certain time points (*n*), 1.5 mL (*Ve*) of supernatant was taken out and replaced with 1.5 mL of fresh PBS. The concentration of Pac-525 (*C_n_*_−1_) was measured using a UV–VIS spectrophotometer at 280 nm, while the concentration of BMP-2 was measured using an ELISA kit. M represents the amount of drug in microspheres in Section 2.3.2. The measurement for each sample group was repeated three times. The cumulative release rate (*En*) was calculated with the following formula:

(3) $$En=\frac{(Ve\times \sum_{1}^{n-1}C_{n-1}+V_{0}\times C_{n})}{M}\times 100\;\%$$

### 2.4. Biological Properties of PLGA_(BMP-2)_/CS_(Pac-525)_@MC/PCL Scaffolds

#### 2.4.1. Cell Culture

Briefly, bone marrow mesenchymal stem cells (BMSCs) were cultured in Dulbecco’s modified Eagle’s medium (DMEM, Gibco Invitrogen Corporation/Life Technologies Life Sciences, Carlsbad, CA, USA) containing 10% fetal bovine serum (FBS, GIBCO Invitrogen Corporation/Life Technologies Life Sciences, Carlsbad, CA, USA) and 100 U/mL penicillin and 100 mg/mL streptomycin [28]. The biocompatibility of the scaffolds was proven through the viability of the BMSCs in the samples with a CCK-8 kit (Beyotime Institute of Biotechnology, Jiangsu, China) and cell morphology. The prepared scaffolds were cut into thin slices and sterilized with 60 Co γ-irradiation at a dose of 15 kGy. Then, 1.5 × 10^4^/mL of cells was implanted on sterilized scaffolds in 48-well plates and incubated at 37 °C and 5% CO_2_.

#### 2.4.2. Cell Adhesion

For SEM observation, the prepared scaffolds with BMSCs were fixed with 2.5% glutaraldehyde in PBS for 2–3 h. After ethanol gradient dehydration, the samples were freeze-dried and sprayed with gold for SEM observation.

For CLSM observation, the BMSCs were cultured on the scaffolds for 48 h. The samples were taken out from the 48-well plate and fixed with 4% (*v*/*v*) paraformaldehyde for 20–30 min. Then, phalloidm-rhodamine (Invitrogen, Carlsbad, CA, USA) was used to stain the cytoplasm of the BMSCs, and DAPI (Sigma-Aldrich Co., LLC., Rockville, MD, USA) was used to stain the nuclei of the BMSCs. The samples were directly observed via CLSM.

#### 2.4.3. Cell Viability

Cell viability was detected with a CCK-8 assay. In brief, BMSCs were cultured in the scaffolds as described above. At the scheduled time points (1, 3, 5 days), the cells were tested with a CCK-8 kit, and the OD value was read using a microplate reader (Bio-Rad, Model 680, Hercules, CA, USA) at 450 nm. All tests were repeated three times.

#### 2.4.4. Detection of Osteogenic Activity

The bioactivity of the BMP-2 released from the microspheres and scaffolds was measured using the alkaline phosphatase (ALP) activity in vitro. After 24 h of culture, the culture medium was replaced with the differentiation medium (DMEM + 10 mmol/L sodium glycerophosphate + 10% FBS + 0.1 mol/L dexamethasone + 50 mg/vitamin C + penicillin and streptomycin), and the supernatant containing the BMP-2 released from the samples was added to the plates. The medium was changed every three days. After 14 days of incubation, BMSCs were lysed with RIPA, and the supernatant was analyzed using the operation of the ALP ELISA kit. The measurement for each sample group was repeated three times. The ALP activity is expressed per microgram (mg) of protein. The total protein in the cell was measured using Pierce™ BCA protein assay kits (Thermo Fisher Scientific Inc., Waltham, MA, USA).

### 2.5. Antibacterial Property of PLGA_(BMP-2)_/CS_(Pac-525)_@MC/PCL Scaffolds

An antibacterial cyclome test was used to evaluate the antibacterial activity of the PLGA_(BMP-2)_/CS_(Pac-525)_@MC/PCL scaffolds at different time points (10, 20, 30 days) against *S. aureus* and *E. coli*. Briefly, *S. aureus* and *E. coli* were cultured in Luria–Bertani (LB) medium at 37 °C overnight. About 1 mL of the sustained-release solution was added to Oxford cups at the scheduled times (10, 20, 30 days). After incubating at 37 °C for 24 h, the size of the inhibition zone was observed to examine the antibacterial activity of the samples.

### 2.6. Statistical Analysis

All of the results are expressed as mean ± standard deviation. The statistical analysis was performed by one-way ANOVA test with SPSS 26.0 software. All data were analyzed at least three times. If *p* < 0.05, the result was considered to be significantly different.

## 3. Results and Discussion

### 3.1. Morphology of Microspheres and Scaffolds

**Figure 1:**
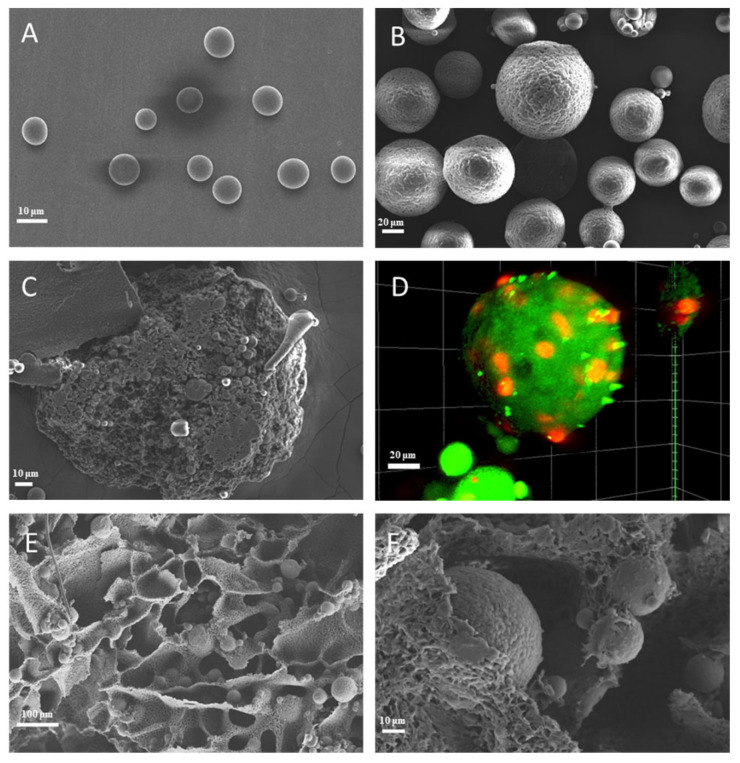
Morphology of microspheres and scaffolds. (**A**) SEM micrographs of PLGA_(BMP-2)_ microspheres; (**B**,**C**) SEM images of PLGA_(BMP-2)_/CS_(Pac-525)_ microspheres ((**B**) surface; (**C**) cross-section); (**D**) CLSM images of PLGA_(Nile)_/CS_(FITC)_ microspheres (PLGA: red; CS: green); (**E**,**F**) SEM images of PLGA_(BMP-2)_/CS_(Pac-525)_@MC/PCL scaffolds.

The PLGA_(BMP-2)_ microspheres prepared by electrospraying were round in shape, with a smooth surface (Figure 1A). The morphology of the PLGA_(BMP-2)_/CS_(Pac-525)_ microspheres was dense and wrinkled, as shown in Figure 1B. And the PLGA_(BMP-2)_ microspheres were warped and dispersed into the CS shell, forming a sphere-in-sphere structure, as shown in the cross-sections of the PLGA_(BMP-2)_/CS_(Pac-525)_ and PLGA_(Nile)_/CS_(FITC)_ microspheres (Figure 1C,D). The composite scaffolds prepared by the freeze-drying method showed an interconnected three-dimensional porous structure that lacked regularity. The PLGA_(BMP-2)_/CS_(Pac-525)_@MC/PCL scaffolds presented a porous structure, in which the composite microspheres were evenly distributed in the scaffold, as shown in Figure 1E. Furthermore, on the surface of the scaffolds, there were more microscale structures on the surfaces of the pore walls, possibly due to the addition of MC (Figure 1F). These different sizes of micro–nano surface structures could have increased the surface roughness of the material and then promoted cell adhesion and differentiation [29].

### 3.2. Physical and Chemical Characteristics

**Figure 2:**
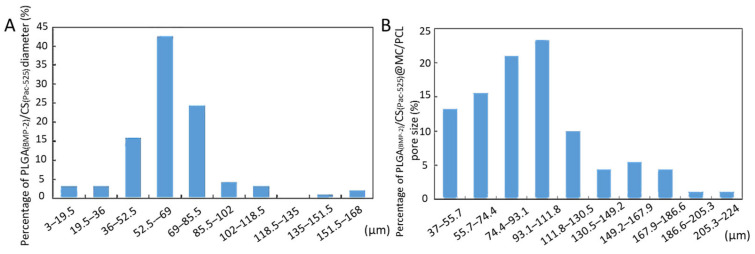
Physical characteristics of microspheres and scaffolds. (**A**) The diameter range of PLGA_(BMP-2)_/CS_(Pac-525)_ microsphere; (**B**) the pore size range of PLGA_(BMP-2)_/CS_(Pac-525)_@MC/PCL scaffolds.

The average particle sizes of the PLGA_(BMP-2)_ microspheres and PLGA_(BMP-2)_/CS_(Pac-525)_ composite microspheres were 8.9 ± 1.0 and 61.26 ± 15.32 μm (Table 1). And the diameter of the PLGA_(BMP-2)_/CS_(Pac-525)_ composite microspheres was mainly distributed in 36–86 μm (Figure 2A). The pore diameter of the scaffold mainly ranged from 74.4 µm to 111.8 µm (Figure 2A), with an average pore diameter of about 97.68 ± 3.43 µm (Table 2).

The Pac-525 encapsulation rate of the chitosan shell was about 66.21 ± 2.29%, and the encapsulation rate of BMP-2 in the PLGA microspheres was 83.75 ± 3.25% (Table 1). The encapsulation efficiency of the PLGA_(BMP-2)_ microspheres prepared by electrospraying was much higher than that of those prepared by the traditional method. The electrospraying technique is more accessible and results in higher entrapment efficiency [30]. The nanoparticles prepared by Qi Wang using the double emulsion loading method had loading contents of 11.6 ± 3.2% (SDS) and 13.8 ± 2.4% (PVA) [31]. Therefore, the electrospraying technique successfully reduced the loss of AMPs during the preparation of the PLGA_(BMP-2)_/CS_(Pac-525)_@MC/PCL scaffolds.

The porosity of the PLGA_(BMP-2)_/CS_(Pac-525)_@MC/PCL scaffolds was 72.75 ± 2.61%. As mentioned in previous research, porous scaffolds are infiltrated by capillaries, perivascular tissues, and osteoprogenitor cells for the formation of newly regenerated bone [32]. The porosity of the PLGA_(BMP-2)_/CS_(Pac-525)_@MC/PCL scaffolds was approximately 70–75%, which is similar to that of trabecular bone (75–85%) [33]. However, excessively high porosity may not be beneficial for maintaining appropriate compressive strength. The compressive strength of the PLGA_(BMP-2)_/CS_(Pac-525)_@MC/PCL scaffolds was 0.422 ± 0.019 MPa (Table 2).

### 3.3. The Drug Release Behaviour in Vitro 

**Figure 3:**
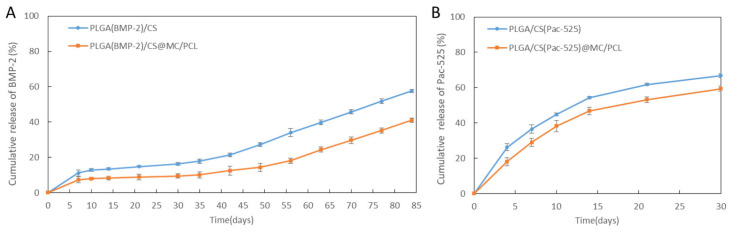
The cumulative release of drugs in microspheres and scaffolds. (**A**) The cumulative release curve of BMP-2 in PLGA_(BMP-2)_/CS microspheres and PLGA_(BMP-2)_/CS@MC/PCL scaffolds; (**B**) the cumulative release curve of Pac-525 in PLGA/CS_(Pac-525)_ microspheres and PLGA/CS_(Pac-525)_@MC/PCL scaffolds.

As shown in Figure 3A,B, the BMP-2 and Pac-525 in the PLGA_(BMP-2)_/CS_(Pac-525)_ microspheres and PLGA_(BMP-2)_/CS_(Pac-525)_@MC/PCL scaffolds both showed rapid release in the early stage in the first 7 days. On the seventh day, the cumulative release rates of BMP-2 and Pac-525 in scaffolds were about 10% and 30%, respectively. The release process of BMP-2 in PLGA microspheres lasted over 85 days, while that of Pac-525 in the CS microspheres was sustained for about 30 days.

As shown in Table 3, after 30 days of incubation, the cumulative release rates of Pac-525 from the PLGA/CS_(Pac-525)_ microspheres and PLGA/CS_(Pac-525)_@MC/PCL scaffolds were 66.85 ± 1.04% and 59.42 ± 1.46%, respectively. On the other hand, the cumulative release rates of BMP-2 from the PLGA_(BMP-2)_/CS microspheres and PLGA_(BMP-2)_/CS@MC/PCL scaffolds ere 16.26 ± 0.84% and 9.45 ± 1.11%, respectively. Compared with the release of drug-loaded chitosan microspheres mentioned in the literature [34], the interface energy interfered with the release behaviors of Pac-525 and BMP-2, which made the drug easy to distribute at the interface of PLGA and CS. The release kinetics related to chitosan microspheres were also influenced by the molecular weight and cross-linking degree of chitosan [35]. The different release rates of BMP-2 and Pac-525 (Table 3) were caused by the differences in the release mechanisms of the PLGA microspheres and CS microspheres. Due to the hydrophilicity of CS, it is easily infiltrated by body fluids, leading to the rapid release of Pac-525 into tissues. At the same time, the release of BMP-2 proceeds with the degradation of PLGA [36]. The drug release kinetics of the BMP-2-loaded PLGA microspheres wrapped in the PLGA_(BMP-2)_/CS microspheres and PLGA_(BMP-2)_/CS@/MC/PCL scaffolds were to a large extent affected by the morphologies of the PLGA_(BMP-2)_/CS microspheres during the process of degradation. For example, the cumulative release rates of the PLGA_(BMP-2)_/CS microspheres and PLGA_(BMP-2)_/CS@/MC/PCL scaffolds were only about 13% and 8%, respectively, in the first 10 days, followed by very slow release due to the presence of the chitosan shell. After 30 days, as the shells of the CS and MC/PCL began to crack and lose their integrity, the PLGA microspheres were exposed, leading to a relatively rapid linear release process. This caused two distinct morphological release profiles for BMP-2 and Pac-525. The vitro release kinetics showed that the presence of the scaffold could slow the drug release rate to a certain extent, as shown by comparing the sustained release of PLGA_(BMP-2)_/CS_(Pac-525)_ microspheres with PLGA_(BMP-2)_/CS_(Pac-525)_@/MC/PCL scaffolds. The release curves in Figure 3A,B suggest that the PLGA_(BMP-2)_/CS_(Pac-525)_@/MC/PCL scaffolds inhibited infection immediately and promoted long-term osteogenesis.

### 3.4. Biocompatibility

**Figure 4:**
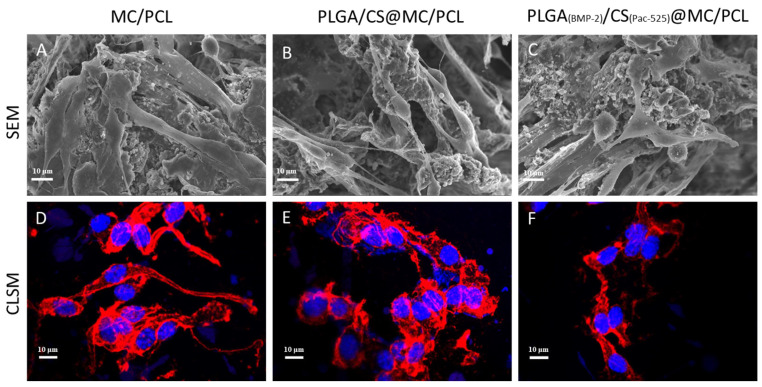
SEM and CLSM images of BMSCs on scaffolds. (**A**,**D**) On MC/PCL scaffolds; (**B**,**E**) on PLGA/CS@MC/PCL scaffolds; (**C**,**F**) on PLGA_(BMP-2)_/CS@MC/PCL scaffolds. (Red: cytoskeleton; Blue: nucleus).

**Figure 5:**
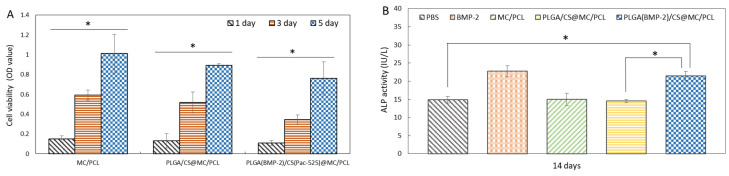
Bioactivity of PLGA_(BMP-2)_/CS@MC/PCL scaffolds. (**A**) BMSCs’ viability on scaffolds on the 1st, 3rd and 5th days; (**B**) ALP activity of BMSCs on scaffolds at 14 days (* *p* < 0.05).

The morphology of the BMSCs adhered to the scaffolds was observed by SEM and CLSM. The BMSCs spread on the surfaces of the PLGA_(BMP-2)_/CS_(Pac-525)_@MC/PCL scaffolds exhibited a well-defined fusiform appearance (Figure 4). Compared to the MC/PCL group, the spread of the cells on the PLGA/CS@MC/PCL and PLGA_(BMP-2)_/CS_(Pac-525)_@MC/PCL scaffolds did not seem to be good enough. Then, the viability of the BMSCs was measured using a CCK-8 kit on the first, third, and fifth days. The number of BMSCs increased over time (Figure 5A). Comparing the cell viabilities on PLGA/CS@MC/PCL and PLGA_(BMP-2)_/CS_(Pac-525)_@MC/PCL scaffolds to those of the MC/PCL group, there was no significant difference (*p* value > 0.05). This suggested that the presence of BMP-2 and Pac-525 did not significantly inhibit cell proliferation. It was inferred that the different morphologies of the cells in the MC/PCL group, and in the PLGA/CS@MC/PCL and PLGA_(BMP-2)_/CS_(Pac-525)_@MC/PCL scaffolds might have been induced by the more coarse surface structure of the composite microspheres. Nevertheless, the function of BMP-2 was not affected by the preparation of the PLGA_(BMP-2)_/CS_(Pac-525)_@MC/PCL scaffolds. The above results indicated that the PLGA_(BMP-2)_/CS_(Pac-525)_@MC/PCL scaffolds had a good biological compatibility

### 3.5. Osteogenic Activity

The osteogenic property of the BMP-2 released from the composite scaffold was determined with an ALP assay kit. As shown in Figure 5B, the ALP activity of the BMSCs in the PLGA_(BMP-2)_/CS@MC/PCL scaffolds was similar to that of the BMP-2 group, which was significantly higher than that of the other groups. The osteogenic property of the PLGA_(BMP-2)_/CS_(Pac-525)_@/MC/PCL scaffolds was significantly improved by BMP-2 in the early stage (Figure 5B) [37]. As demonstrated by the sustained release of BMP-2 in Figure 3A, the PLGA_(BMP-2)_/CS_(Pac-525)_@MC/PCL scaffolds continuously played a positive role in long-term osteogenesis.

### 3.6. Antimicrobial Activity

**Figure 6:**
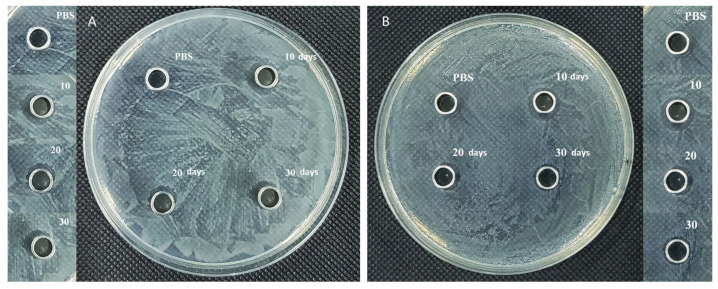
The antibacterial activity of PLGA_(BMP-2)_/CS_(Pac-525)_@MC/PCL scaffolds. The antibacterial activity of the released solution on *E. coli* (**A**) and *S. aureus* (**B**).

In addition to promoting osteogenesis, the antibacterial property of the PLGA_(BMP-2)_/CS_(Pac-525)_@/MC/PCL scaffolds was achieved through the incorporation of Pac-525. As shown in Figure 6, the Pac-525 solution released from the scaffolds for 10, 20, and 30 days was used to treat *S. aureus* and *E. coli* for 24 h. The antimicrobial activity of the Pac-525 released from the composite scaffold against *S. aureus* and *E. coli* was assessed using an antibacterial cyclome test. By observing the size of the antibacterial cyclome, it was found that the samples at 10 days exhibited obvious antibacterial activity. However, compared to the 10-day samples, the diameters of the antibacterial cyclome on both *S. aureus* and *E. coli* were much reduced at 20 days. The antibacterial cyclome diameters of the PLGA_(BMP-2)_/CS_(Pac-525)_@MC/PCL scaffolds time-dependently decreased (Table 4). Pac-525 exhibited excellent antibacterial activity against *Porphyromonas gingivalis*, *Fusobacterium nucleatum*, *S. aureus,* and *E. coli* [38]. An antibacterial cyclome formed, indicating the long-term antibacterial activity of the PLGA_(BMP-2)_/CS_(Pac-525)_@MC/PCL scaffolds. The formation of a clear zone around the Oxford cup indicated that the antibacterial property of Pac-525 was not affected by the preparation process. As demonstrated by the sustained release of Pac-525 shown in Figure 3B, the antibacterial function of Pac-525 mainly happened in the first 30 days. Then, the function of the PLGA_(BMP-2)_/CS_(Pac-525)_@MC/PCL scaffolds was used to promote bone regeneration. To address the problem of resistance to traditional antibiotics, many agents such as metal ions [39,40], lysosomes [41], antimicrobial peptides, and so on, have been investigated. Currently, the mechanism of action of AMPs is still unclear. AMPs might inherently structure with the target and interact with the lipid membrane of the microorganism through electrostatic interactions and receptor-mediated membrane interactions [42,43]. In our previous research, targeting the LPS on Gram-negative bacteria might be one antibacterial mechanism of Pac-525 [18]. Overall, the PLGA_(BMP-2)_/CS_(Pac-525)_@MC/PCL scaffolds could maintain their antibacterial activity thanks to Pac-525.

## 4. Conclusions

In summary, the prepared PLGA_(BMP-2)_/CS_(Pac-525)_@MC/PCL scaffolds comprised MC/PCL porous scaffolds and PLGA_(BMP-2)_/CS_(Pac-525)_ microspheres. The PLGA_(BMP-2)_/CS_(Pac-525)_ composite microspheres prepared with electrospraying and crosslinking emulsion technology realized the dual-stage release of Pac-525 and BMP-2, providing dual antibacterial and osteogenesis functions. The composite scaffold had a three-dimensional porous structure and good osteogenic properties. Meanwhile, the PLGA_(BMP-2)_/CS_(Pac-525)_@MC/PCL scaffolds provided long-term antibacterial activities on both Gram-positive and -negative bacteria in vitro. In a future study, the application of PLGA_(BMP-2)_/CS_(Pac-525)_@MC/PCL scaffolds in vivo will be carried out.

## Figures and Tables

**Table 1:** Diameter and encapsulation ratio of microspheres.

Name	Mean Diameter (µm)	Encapsulation Ratio (%)
PLGA_(BMP-2)_	8.9 ± 1.0	83.75 ± 3.25
PLGA/CS_(Pac-525)_	--	66.21 ± 2.29
PLGA_(BMP-2)_/CS_(Pac-525)_	61.3 ± 15.3	--

**Table 2:** Physical characteristics of scaffolds.

Name	Average Pore Size (µm)	Porosity (%)	Compressive Strength (Mpa)
PLGA_(BMP-2)_/CS_(Pac-525)_@MC/PCL	97.7 ± 3.4	72.75 ± 2.61	0.42 ± 0.02

**Table 3:** Cumulative release rate of microspheres and scaffolds over 30 days.

Cumulative Release Rate (%)	Microsphere		Scaffold	
	PLGA_(BMP-2)_/CS	PLGA/CS_(Pac-525)_	PLGA_(BMP-2)_/CS@MC/PCL	PLGA/CS_(Pac-525)_@MC/PCL
BMP-2	16.26 ± 0.84	--	9.45 ± 1.11	--
Pac-525	--	66.85 ± 1.04	--	59.42 ± 1.46

**Table 4:** The antibacterial cyclome diameters on *E. coli* and *S. aureus* (^#^ fold change compared with PBS).

Name	PBS	10 Days ^#^	20 Days ^#^	30 Days ^#^
*E. coli*	1 ± 0.02	1.57 ± 0.10	1.36 ± 0.04	1.30 ± 0.04
*S. aureus*	1 ± 0.02	1.35 ± 0.01	1.14 ± 0.02	1.15 ± 0.03

## Data Availability

Data are contained within the article.
